# Anomalous Oligomerization Behavior of *E. coli* Aquaporin Z in Detergent and in Nanodiscs

**DOI:** 10.3390/ijms24098098

**Published:** 2023-04-30

**Authors:** Wahyu Surya, Clare Pei Yii Yong, Anu Tyagi, Shashi Bhushan, Jaume Torres

**Affiliations:** School of Biological Sciences, Nanyang Technological University, 60 Nanyang Drive, Singapore 637551, Singapore

**Keywords:** aquaporin, nanodiscs, electron microscopy, oligomerization, mass photometry, analytical ultracentrifugation

## Abstract

Aquaporins are tetrameric integral membrane proteins that act as water channels, and can also permeabilize membranes to other solutes. The monomer appears to be the functional form despite all aquaporins being organized as tetramers, which therefore must provide a clear functional advantage. In addition to this quaternary organization, some aquaporins can act as adhesion molecules in membrane junctions, when tetramers located in opposing membranes interact via their extracellular domains. These stacked forms have been observed in a range of aquaporins, whether using lipidic membrane environments, in electron crystallography, or using detergent micelles, in single-particle cryo-electron microscopy (cryo-EM). In the latter technique, structural studies can be performed when the aquaporin is reconstituted into nanodiscs of lipids that are surrounded by a protein scaffold. During attempts to study *E. coli* Aquaporin Z (AqpZ), we have found that in some conditions these nanodiscs tend to form filaments that appear to be either thicker head-to-tail or thinner side-to-side stacks of nanodiscs. Nanodisc oligomerization was observed using orthogonal analytical techniques analytical ultra-centrifugation and mass photometry, although the nature of the oligomers (head-to-tail or side-to-side) could not be determined. Using the latter technique, the AqpZ tetramer itself formed oligomers of increasing size when solubilized only in detergent, which is consistent with multiple stacking of AqpZ tetramers. We observed images consistent with both of these filaments in negative staining EM conditions, but only thicker filaments in cryo-EM conditions. We hypothesize that the apparent nanodisc side-to-side arrangement that can only be visualized in negative staining conditions is related to artifacts due to the sample preparation. Filaments of any kind were not observed in EM when nanodiscs did not contain AqpZ, or after addition of detergent into the nanodisc cryo-EM preparation, at concentrations that did not disrupt nanodisc formation. To our knowledge, these filaments have not been observed in nanodiscs preparations of other membrane proteins. AqpZ, like other aquaporins has a charge asymmetry between the cytoplasmic (more positive) and the extracellular sides, which may explain the likely head-to-tail stacking observed, both in nanodisc preparations and also in detergent micelles.

## 1. Introduction

Aquaporins (AQPs) constitute a family of tetrameric integral membrane proteins (IMPs) that permeabilize membranes to water and other solutes [1]. In particular, *E. coli* Aquaporin Z (AqpZ) permeabilizes cellular membranes to water with high selectivity [1,2,3,4] and is a workhorse for the fabrication of biomimetic membranes for water purification [5,6,7,8,9]. Despite differences in physiological features, selectivities, and permeabilities, all aquaporins oligomerize as homotetramers, as shown by the current 58 AQP structures in the Protein Data Bank (as of April 2023). The monomer, however, is the water channel functional unit [10,11], and the reason for a tetrameric structure is not well understood. Tetramerization provides higher stability, and it is likely that monomer function is tightly coordinated with the other monomers in the tetramer [12,13,14]. In addition to this tetrameric organization, some aquaporins have been found to participate in junctions between adjacent membranes; tetramers in opposite membranes interact with each other via interactions mediated by their extracellular domains, thus acting as adhesion molecules [15,16,17,18,19,20,21,22,23]. Such symmetric head-to-head interaction has been observed when aquaporin is embedded in detergent for single-particle cryo-electron microscopy (cryo-EM) methods. For example, the structure of AQP7 was recently obtained using single-particle cryo-EM after solubilization of aquaporin in glyco-diosgenin detergent (GDN) [23], where it formed dimers of tetramers. Other aquaporins have been previously shown to produce similar interactions in lipid environments using electron crystallography [24]. Clearly, a lipid environment is more desirable as it better represents the native membrane. Nanodiscs constitute another system where the target IMP can be incorporated into a lipid environment of choice, but at a relatively high lipid-to-protein ratio [25].

We describe herein an anomalous behavior observed for *E. coli* AqpZ when it is reconstituted in a mixture of synthetic lipids in nanodiscs, and also when solubilized in detergent micelles. We have used electron microscopy, analytical ultracentrifugation and mass photometry to characterize this behavior. To the best of our knowledge, similar oligomerization behavior has not been described for other IMPs inserted into nanodiscs [26]. It is possible that this is a unique feature of AqpZ and other similar aquaporins, facilitated by their small extramembrane domains and the opposite electric charge distribution between cytoplasmic and extracellular sides.

## 2. Results

### 2.1. Nanodisc Formation

**Figure 1:**
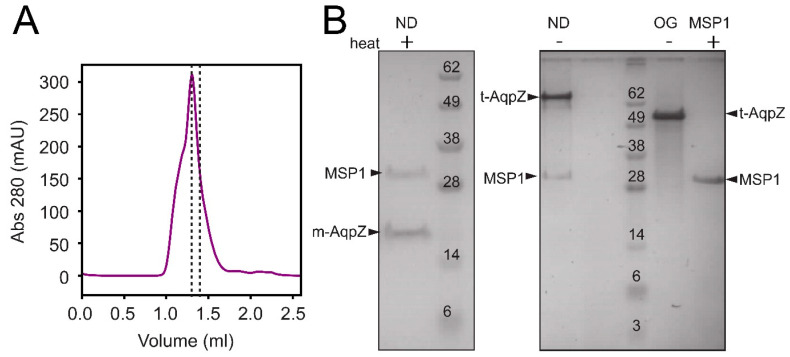
Assembly and purification of AqpZ nanodiscs. (**A**) Size exclusion chromatogram of AqpZ nanodiscs assembled from AqpZ, MSP1E3D1 (MSP1) and DOPC/POPG (4:1 molar ratio). The collected fraction is indicated between dotted lines; (**B**) SDS-PAGE of purified AqpZ nanodiscs (ND), either heated to 70 °C (+) or unheated (−). AqpZ migrates as a monomer (m-AqpZ) when heated, and as a tetramer (t-AqpZ) when not heated. Reference lanes labeled ‘OG’ and ‘MSP1′ are AqpZ in OG micelles and purified MSP1, respectively.

Aquaporin Z was first assembled into nanodiscs (Figure 1, labeled ND), where it was exposed to a negatively charged lipid membrane (DOPC/POPG) in order to mimic the conditions found in the *E. coli* membrane. The chromatogram of the elution (Figure 1A) shows the fraction (dotted lines) used in further experiments. This fraction consisted of a homogeneous preparation of nanodiscs containing both MSP1 and AqpZ (Figure 1B, left panel), where the latter is monomeric (m-AqpZ) after heating. The right panel in Figure 1B shows that when the sample is not heated, AqpZ appears as a tetramer (t-AqpZ). This behavior unequivocally identifies the AqpZ component unequivocally. In unheated AqpZ in detergent OG, t-AqpZ can also be observed, although moving slightly faster than in sample ND. This effect may be caused by the presence of bound lipids to AqpZ in the latter sample, which may modify SDS binding. As expected, no bands corresponding to m-AqpZ or t-AqpZ are observed when only MSP1 is used.

### 2.2. Nanodisc Homogeneity Using AUC

**Figure 2:**
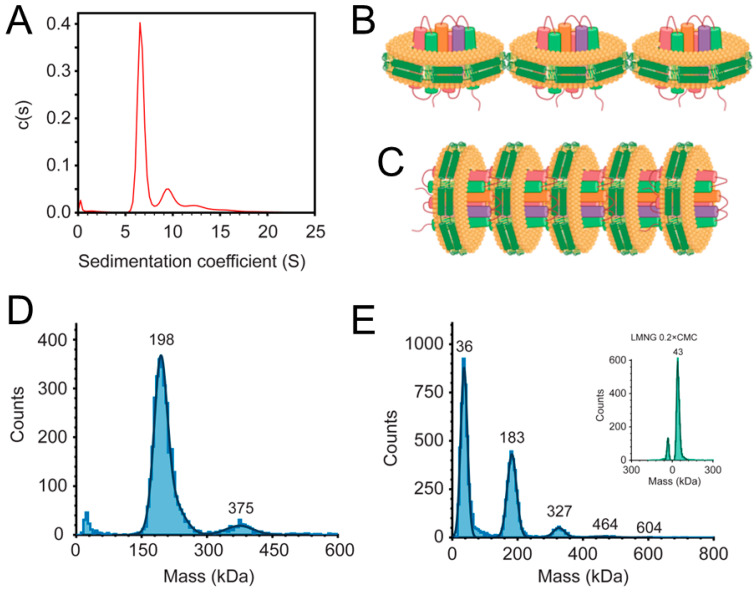
Mass/size measurements of AqpZ nanodiscs. (**A**) Sedimentation coefficient distribution of AqpZ nanodiscs obtained by the AUC-SV analysis in 300 mM of NaCl; (**B**,**C**) schematics corresponding to hypothetical AqpZ-containing nanodisc arrangements in side-to-side (**B**) and head-to-tail (**C**) arrangements. Cartoon drawings were created with BioRender; (**D**) mass photometry measurement of AqpZ nanodiscs in 300 mM of NaCl; (**E**) AqpZ in LMNG detergent (0.2 × CMC) in 50 mM of NaCl. Inset shows the histogram for LMNG detergent alone at 0.2 × CMC as a reference for the detergent noise peak at approximately 40 kDa.

The homogeneity of the nanodiscs preparation was tested using AUC-SV (Figure 2A). This figure shows that the majority of the sample sedimented with an S value of about 6.5 S (~80% of the sample), but faster species can also be observed at 9.5 S, 12.5 S and even at 15.5 S. Multiple bands in a c(s) plot can indicate the undesirable presence of nanodiscs of multiple sizes, or incompletely solubilized liposome particles. However, it is also possible that the preparation is homogeneous but faster particles represent the aggregation of individual nanodiscs into multimeric forms. The next band (9.5 S) was less than double the sedimentation coefficient of the first band (6.5 S), despite the doubling of the mass in the resulting particle. In principle, a dimer of nanodiscs in a side-to-side form (Figure 2B) would oppose more friction than a face-to-tail form (Figure 2C), and would therefore have a lower S value. To confirm this, we used a nanodisc model in the PDB (6CLZ) (see Materials and Methods section). After removing the lipid binding protein, the particle was formed by two copies of Apo-A1 and 218 molecules of DMPC. In Hydropro, the S value of the single nanodisc was predicted to be 1.98 S, whereas the S value for face-to-tail and side-to-side dimers was less than double in both cases: 3.16 S and 2.82 S, respectively. Thus, the S value that was experimentally obtained in the AUC does not necessarily represent a side-to-side interaction and can equally be caused by a face-to-tail interaction.

### 2.3. Mass Photometry

**Figure 3:**
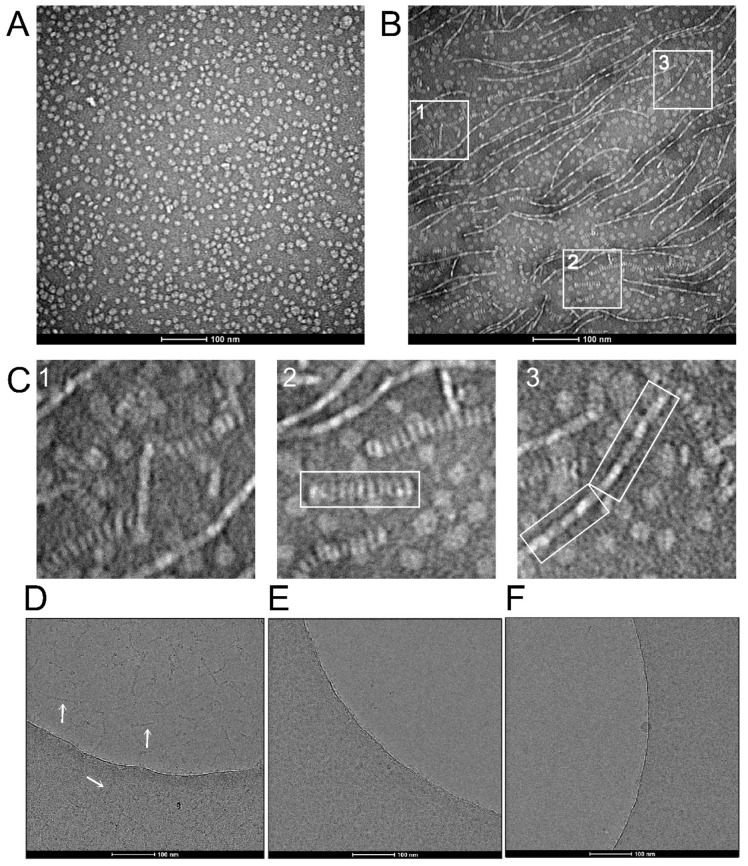
EM micrographs of AqpZ nanodiscs. (**A**) Electron micrograph of negatively stained AqpZ nanodiscs showing homogeneously distributed particles in 300 mM of NaCl; (**B**) same as (**A**), taken from a different part of the grid and showing fibrillar and rouleaux formations; (**C**) selected regions from (**B**) (squares of 123 × 123 nm), showing a close-up of nanodiscs fibers, referred to as head-to-tail (panel 2) or side-to-side (panel 3) filaments; (**D**) cryo-electron micrograph of AqpZ nanodiscs in 300 mM of NaCl, showing thick filaments in both ice and carbon areas (arrows); (**E**) same as (**D**), but in the presence of 0.1% OG, where the filaments disappeared and individual nanodiscs can be seen in the carbon area; (**F**) micrograph of ‘empty’ nanodiscs in the absence of added detergent, where individual nanodiscs can be seen in the carbon area.

To clarify this issue, the same nanodisc sample was analyzed by mass photometry. The majority of the sample appeared as a band centered at 198 kDa (Figure 2D) which likely represents a single nanodisc. Indeed, the molecular weight of the AqpZ tetramer is 100 kDa (the monomer is 23,702 Da, plus the His-tag), and the MSP fraction is ~65 kDa (i.e., a dimer of the 32.7 kDa monomer), therefore the lipid fraction must represent approximately 35 kDa. The latter is consistent with the approximate dimensions of these nanodiscs. The expected size is 10 nm in diameter, and also determined by EM (see Figure 3 below). Assuming that the diameter occupied by the lipid fraction is approximately 8 nm (considering that the MSP1 barrier is 1 nm thick), this corresponds to a circular area of 50 nm^2^. Once subtracted the area occupied by the AqpZ tetramer (36 nm^2^, PDB, 1RC2), the remaining 14 nm^2^ are occupied by lipids. Using a cross-sectional area of the lipids of 0.50–0.65 Å^2^ [27,28], the number of lipid molecules in the nanodisc is ~50 (i.e., 25 for each leaflet, also consistent with the reconstitution ratio AqpZ:MSP1E3D1:lipid = 4:2:40). If the average molecular weight of the lipids used is 0.8 kDa, this represents 40 kDa, close to the 35 kDa obtained from mass photometry for a single nanodisc.

An additional band can be observed in Figure 2D, at approximately double the molecular weight (375 kDa). This band probably corresponds to the aggregation of two nanodiscs. An even smaller band could be observed at ~600 kDa, although this was at the limit of sensitivity of the instrument (not shown). Thus, this result is consistent with the multiple bands observed under AUC-SV. However, since mass photometry does not provide information on the shape of the particles, whether these oligomers of nanodiscs involve side-to-side or head-to-tail interactions is not clear. Nevertheless, these data are consistent with the faster bands observed in AUC (Figure 2A) corresponding to aggregation of individual nanodiscs rather than being a part of a heterogeneous mixture of larger nanodiscs or poorly solubilized liposomes.

We then hypothesized that if the interaction occurs through the AqpZ tetramer itself, in a head-to-tail form (Figure 2C), it should also be possible to observe this behavior in detergent micelles using mass photometry (Figure 2E). For this purpose, we used lauryl maltose neopentyl glycol (LMNG) detergent, which has a very low CMC (10 µM) and off-rate that is orders of magnitude lower than that of dodecyl maltoside (DDM) [29,30]). Both a low CMC and slow off-rate are necessary because in mass photometry the concentration of protein must be very low (100 pM–100 nM), therefore this limits the detergent concentrations that can be used. In the histogram of mass distribution, the left-most band (36 kDa) corresponds to detergent micelles without protein. This assignment is made based on a control sample that contained just detergent (see Figure 2E, inset). The next band has a molecular weight of 183 kDa, and likely corresponds to an AqpZ tetramer solubilized in a detergent micelle. Since the molecular weight of tetrameric AqpZ is ~100 kDa and ‘empty’ micelles are 36 kDa, this suggests that the solubilization of an AqpZ tetramer requires about twice the amount of detergent than is required for an empty micelle (i.e., 83 kDa). Other bands corresponding to larger masses were observed, but they did not increase in multiples of 183 kDa: 327, 464, and 604 kDa. We tentatively assign these bands to double, triple and quadruple tetramers. The increment in molecular weight between these species is constant within the limits of the mass error of this technique (±5%), i.e., 144, 137, and 140 kDa, respectively. Thus, assuming a 140 kDa increment, every addition of one tetramer to the complex requires additional 40 kDa of detergent. For example, if the heaviest particle (604 kDa) corresponds to four AqpZ tetramers (400 kDa), it requires 200 kDa of detergent, instead of the total mass of four AqpZ-containing micelles (732 kDa). This suggests that the particles are not just aggregates of micelles, but an ensemble of AqpZ tetramers, possibly head-to-tail (Figure 2C), solubilized by detergent, because a symmetric head-to-head aggregation would likely only produce dimers of AqpZ tetramers. Of course, even heavier species than the ones shown here may be present in even smaller quantities, but this is beyond the sensitivity of the technique (<1 ng).

### 2.4. Electron Microscopy

To determine if the oligomerization of AqpZ-containing nanodiscs is side-to-side or head-to-tail (Figure 2B,C), we used an EM analysis (Figure 3). The preparation of nanodiscs was found to be very homogeneous, as shown using negative staining, with nanodisc diameters of about 10 nm (Figure 3A). However, in other parts of the sample, we could observe fibrillar structures consistent with aggregation of nanodiscs (Figure 3B). These structures are clearly reminiscent of the nanodisc oligomerization behavior observed in AUC (Figure 2A) and in mass photometry (Figure 2D). A magnified image of three areas (labeled 1–3) in Figure 3B shows two types of filaments (Figure 3C). In particular, a thicker structure similar to a stack of coins is highlighted in panel 2, whereas thinner fibers, more consistent with a side-to-side interaction of the nanodiscs, are highlighted in panel 3. Although the scaffold protein (MSP) may play a role in these interactions, these likely mainly involve AqpZ, since oligomerization of AqpZ tetramers was also present in detergent micelles which do not have an MSP component (Figure 2E). We found that in negative staining, these fibers were present without significant changes in three salt concentrations, 50, 300 and 500 mM (not shown).

To confirm that these structures were not artefacts due to the negative staining procedure, we performed similar experiments using cryo-EM (Figure 3D). Similar head-to-tail stack structures were observed using this technique in both ice and carbon regions (see arrows), but the thinner side-to-side ones were not observed. However, when the sample was exposed to a sub-CMC concentration of OG detergent (0.1%) (Figure 3E), these structures disappeared and only individual nanodiscs remained. This head-to-tail stack structure, also known as a rouleaux formation, is commonly observed in the negatively stained preparation of human apolipoproteins [31], among which ApoA1 is the precursor of MSP1 that forms the nanodiscs [25]. To confirm that this behavior is mediated by AqpZ and not by the scaffold protein or the lipid component, we repeated the experiment using empty nanodiscs with the same lipid composition. No filament appeared in any region of the image, which indicates that this behavior is linked to the AqpZ component (Figure 3F).

**Figure 4:**
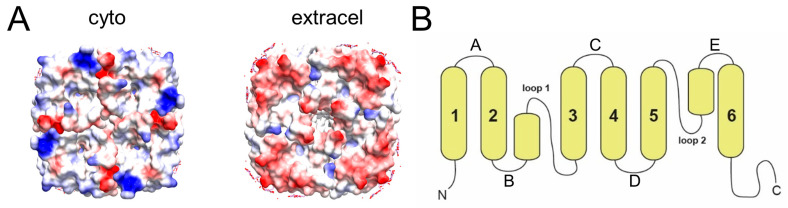
Proposed rationale for nanodisc interaction. (**A**) Cytoplasmic (**left**) and extracellular (**right**) face of AqpZ represented as electric charge (blue = positive; red = negative); (**B**) topology of aquaporins, with six regular helices and loops A, C, and E in the extracellular side.

The head-to-tail stacks interactions may be facilitated by electrostatic interactions. While the two extramembrane domains of AqpZ have a similar pattern of hydrophobicity (not shown), it is clear that there is an asymmetry in the electric charge, more positive in the cytoplasmic side and more negative in the extracellular side (Figure 4A). This may favor a head-to-tail fashion interaction, and may be also responsible for the increasing size of oligomers observed in the presence of detergent (Figure 2E).

**Figure 5:**
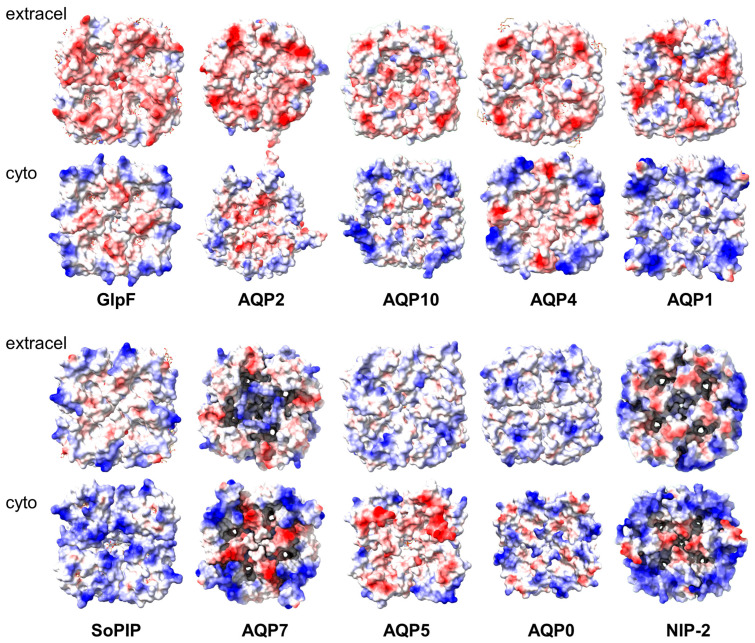
Electric charge distribution in the extra membrane domain of several aquaporins. The structures were obtained from the PDB database and represented in ChimeraX according to electric charge (positive = blue; negative = red). The aquaporins and their PDB codes are GlpF (1FX8), AQP2 (4NEF), AQP10 (6F7H), AQP4 (3GD8), AQP1 (4CSK), SoPIP (3CN5), AQP7 (8AMX), AQP5 (5DYE), AQP0 (1SOR), and NIP-2 (7CJS).

A similar asymmetry is also observed in other aquaporins (Figure 5, top row), although some, e.g., AQP4, are known to only form interactions via the extracellular domain [15]. Thus, this similar charge is not sufficient to prevent face-to-face interactions. In AQP5, the asymmetry is reversed (Figure 5, lower row). It would be interesting to see whether multiple stacks similar to those observed for AqpZ are also observed for these aquaporins. In contrast, they may not be observed for aquaporins that display similar charges on both sides, e.g., the spinach aquaporin SoPIP2 or AQP7.

## 3. Discussion

In aquaporins, the role of the interaction between the monomers in the tetramer is still not clear. Based on a 3.2 Å crystal structure [12], it was proposed that asymmetrical interactions between the AqpZ monomers regulate the open probability of the AqpZ channel via the stabilization of either of two distinct conformations of the side chain of residue Arg-189. Native mass spectrometry experiments in the gas phase have shown that cardiolipin (CDL) in particular, but also other lipids, tightly bind AqpZ [13], contributing to both AqpZ stabilization and more than doubling the water flux. Consistent with this, we showed that the permeability of AqpZ reconstituted in synthetic dioleoyl-glycero-phosphocholine (DOPC) membranes was ~60% lower than after supplementation with CDL, and about 40% lower than when using native *E. coli* membranes [14]. In the absence of externally added CDL, AqpZ that was reconstituted in DOPC lipids lost its α-helical structure after one month of storage at 4 °C, but AqpZ was still α-helical when supplemented with CDL. The concentration-dependence of CDL was sigmoidal, suggesting a cooperative interaction between the monomers in the tetramer.

The stability of the tetrameric form has been found to be important in the function of other aquaporins. For example, heterotetramers can regulate AQP function in plant AQPs [32]. Furthermore, tetramerization may have the advantage of generating a central pore, which in AQP1 was suggested to transport carbon dioxide and cations [33,34]. Tetramerization has also been linked to the ability of AQPs to relocalize to the plasma membrane, e.g., in AQP2, which localizes to the apical membrane of the collecting duct in the mammalian kidney, and examples exist for other AQPs [35,36,37]. It has been speculated that these trigger-induced relocalization responses involve interactions with proteins that only recognize the tetrameric form of AQPs. For AQP4, it was shown that mutations in an intracellular loop (loop D, see Figure 4B) reduced tetrameric oligomerization [38], which led to a lack of relocalization to the plasma membrane in response to changes in extracellular tonicity. Interestingly, human AQP3 was reported to exist in all four possible oligomeric states (monomer, dimer, trimer, and tetramer) in the plasma membrane of erythrocytes [39]. Further, the S-nitrosylation of AQP11 at a cysteine residue in the extracellular loop E (Cys227) has been suggested to be required for AQP11 oligomeric assembly [40], supporting the idea of a role for post-translational modification in AQP oligomerization.

The oligomerizing behavior observed herein for AqpZ is mildly reminiscent of the behavior of other AQPs, such as AQP4 and AQP0, which are involved in tight junctions (see [15] for a review). AQP0 is the most abundant membrane protein in lens fiber cells [16], is found enriched in thin junctions as orthogonal arrays [17] and its reconstitution into liposomes causes the clustering of vesicles [41]. Two-dimensional (2D) crystallization experiments resulted in single- and double-layered crystals [18,19]. As fiber cells of the lens cortex get older, a fraction of AQP0 is proteolytically cleaved [42] and leads to array formation [43,44,45]. These double-layered 2D crystals made it possible to determine the structure of the junctional AQP0 [24,46]. In AQP0, the tetramers in the two membrane layers are exactly in register, and interactions between tetramers occur via extracellular loops C and A (Figure 4B). The fact that AQP0 is not glycosylated and that it has a shortened extracellular loop A [45] allows for such interactions.

AQP4 is the main aquaporin in the brain [47,48], and in glial cells it forms orthogonal arrays [20]. Two splicing isoforms are found, one starting with the first methionine (AQP4M1) and another resulting from cleavage at Met23 (AQP4M23) [49,50]. Similar to AQP0, it is only the truncated form that assembles into orthogonal arrays [51], and tetramers in adjacent membranes interact via loop C [21]. However, AQP4 differs from AQP0 in that a tetramer in one membrane is at the center of four tetramers in the adjoining membrane. The physiological role of AQP4 junctions may be related to osmo-regulation [21]. SoPIP2;1, found in spinach leaf plasma, also forms double-layered 2D crystals upon reconstitution [22]. Recently, a single-particle cryo-EM structure of aquaglyceroporin 7 (AQP7) determined at 2.55 Å resolution has also been found to adopt the form of two adhering tetramers [23]. AQP7 facilitates glycerol flux across the plasma membrane in human pancreatic α- and β- cells, and the interaction between tetramers is also mediated by extracellularly exposed loops. As in AQP0 or AQP4, interactions between the two AQP7 tetramers involve their C loops.

However, in these examples, aquaporins interact symmetrically through the same domains, i.e., in head-to-head (or tail-to-tail) fashion. In contrast, the long stacks that we observe for AqpZ are unlikely to be explained by such symmetric interaction because that would end up in the formation of self-limiting dimers of tetramers, like those observed in AQP0 or AQP7. Of course, long stacks could still be explained by symmetric interactions if there was a similar affinity for interactions at either side of the nanodisc. However, the fact that both sides would interact with partners of similar electric charge composition makes this scenario unlikely. It is worth noting that previous studies using AUC-SV of empty nanodiscs consisting of only MSP1D1 and POPC/POPG lipid mixture did not show any oligomers [52]. This, together with the observation of multiple oligomers of AqpZ in detergent micelles, indicates that the behavior we observe is only attributable to AqpZ. To the best of our knowledge, similar oligomerization behavior has not been described for other IMPs when inserted into nanodiscs [26]. It is possible that this is a feature that may be encountered in other aquaporins that have similar charge asymmetry (Figure 5). Alternatively, a specific motif may be responsible for this interaction in AqpZ.

Other proteins have been reported to form head-to-tail filaments in vitro, such as VEL domains in Arabidopsis plants. VEL domains contain a four-helix bundle that is responsible for spontaneous head-to-tail polymerization. This results into condensates that form intertwined protofilaments in cell nuclei [53]. Other head-to-tail polymerization folds are the sterile alpha motif (SAM) and disheveled and axin (DIX) domains, which mediate the assembly of the condensates that facilitate transduction, transcription, and RNA processing [54,55,56,57]. In all of these cases, polymerization can be blocked by specific point mutations that inhibit condensation in cells [58,59].

Overall, whether a specific or non-specific interaction—mediated by opposing charges—is responsible for the oligomerization observed in AqpZ is not clear. It is also unknown if this behavior has biological significance in *E. coli*. Alphafold-2 could not identify any head-to-tail interaction when we used eight AqpZ monomers as input (not shown), which may suggest that the interaction does not involve specific motifs.

## 4. Materials and Methods

### 4.1. Assembly and Purification of AqpZ Nanodiscs

Aquaporin Z from *E. coli* (AqpZ) was expressed and purified by anion exchange and metal-affinity chromatography as previously described [60]. The plasmid-expressing Membrane Scaffold Protein 1 construct E3D1 (MSP1E3D1) was a gift from Stephen Sligar (Addgene plasmid #20066; http://n2t.net/addgene:20066 (accessed on 15 January 2023); RRID: Addgene_20066) [61]. Protein MSP1E3D1 was expressed and purified as described [62]. AqpZ was reconstituted into nanodiscs by mixing AqpZ, MSP1E3D1, and the lipid mixture 1,2-dioleoyl-sn-glycero-3-phosphocholine/1-palmitoyl-2-oleoyl-sn-glycero-3-phospho-(1′-rac-glycerol) (DOPC/POPG, 4:1 molar ratio) (Avanti Polar Lipids, Alabaster, AL, USA) at a 4:2:40 molar ratio, respectively, in the presence of 1% (*w*/*v*) octylglucoside (OG) (CMC = 0.53%) (Anatrace, Maumee, OH, USA) in 40 mM of Tris, pH 8, 1 mM of EDTA, and 300 mM of NaCl (i.e., TEN buffer). The concentration of AqpZ was 30 µM in all samples except in the sample for the cryo-EM analysis, where it was 120 µM. The mixture was incubated for 1 h and dialyzed overnight in TEN buffer. AqpZ nanodiscs were purified using size exclusion chromatography in TEN buffer using a Superdex 200 Increase 3.2/300 column (Cytiva). The sample with empty nanodiscs (no AqpZ) was prepared by mixing 15 µM of MSP1E3D1 and 1.2 mM of the DOPC/POPG lipid mixture in the same buffer and detergent conditions. Incubation, dialysis, and purification steps were the same as those followed for AqpZ-containing nanodiscs.

### 4.2. Analytical Ultracentrifugation (AUC)

AUC sedimentation velocity (AUC-SV) experiments were performed on a Beckman ProteomeLab XL/I analytical ultracentrifuge (Beckman Coulter, Brea, CA, USA) with An-50 Ti analytical rotor. AqpZ nanodiscs at 2 µM in TEN buffer were loaded into a 2-sector AUC cell with an Epon centerpiece and centrifuged at 35,000 rpm at 20 °C. Absorbance data at 280 nm were collected every 10 min for 15 h. The data were fitted to a c(s) distribution model in SEDFIT [63] and plotted in GUSSI [64] using buffer density = 1.01175 g/mL, buffer viscosity = 1.0412 cP (calculated using SEDNTERP [65]), and partial specific volume of AqpZ nanodiscs = 0.787 mL/g (calculated from the composition). Nanodiscs models consisting of a single nanodisc, and two nanodiscs arranged head-to-tail or side-by-side were derived from the PDB structure of MT1-MMP HPX domain bound to ApoA1-DMPC nanodiscs (Accession ID: 6CLZ) by removing the MT1-MMP HPX protein and duplicating the model in PyMOL (The PyMOL Molecular Graphics System, Version 2.5.2 Schrödinger, LLC [66]). The sedimentation size of nanodiscs was estimated using Hydropro software [67] using buffer density = 1.01175 g/mL, buffer viscosity = 1.0412 cP (calculated using SEDNTERP [65]) and partial specific volume of nanodiscs = 0.931 mL/g (calculated from the composition).

### 4.3. Mass Photometry

Particle mass measurement was performed using mass photometry using a Refeyn Two^MP^ instrument (Refeyn Ltd., Oxford, UK). The sample of AqpZ nanodiscs consisted of 10 nM AqpZ nanodiscs in TEN buffer, whereas the sample of AqpZ in lauryl maltose neopentyl glycol (LMNG, Anatrace) detergent was prepared at a concentration of 100 nM of AqpZ and 20 µM of LMNG (i.e., 2 × CMC) in 50 mM of HEPES at pH 7.4, 1 mM of EDTA, and 50 mM of NaCl (referred to as HEN buffer). This sample was diluted 10 times with HEN buffer immediately before the measurement to a final concentration of 10 nM of AqpZ and 2 µM of LMNG). The sample of LMNG detergent alone (used as a reference to determine the detergent peak) consisted of 20 µM of LMNG (2 × CMC) in HEN buffer, and it was diluted 10 times with HEN buffer immediately before measurement.

### 4.4. Electron Microscopy

For negative staining, the sample of AqpZ nanodiscs was diluted to 10 µg/mL with TEN buffer. A volume of 4 µL was applied onto a carbon-coated 400-mesh copper grid (EMS) that was glow-discharged in air for 60 s. Grids were blotted from the edge with Whatman grade 1 filter paper and negatively stained with 2% uranyl acetate. The grids were viewed at 68,000× magnifications under an FEI Tecnai T12 transmission electron microscope operating at 120 kV, which was equipped with an Eagle 4K CCD camera. For the cryo-EM analysis, a volume of 4 µL of nanodiscs containing AqpZ at 0.5 mg/mL in TEN buffer was applied onto a Quantifoil 300 mesh Cu R1.2/1.3 holey carbon grid (EMS) that had been glow-discharged in air for 60 s. Grids were blotted at 4 °C and 100% humidity before being plunge-freezed in liquid ethane using the FEI Vitrobot Mark IV plunge freezer. The grids were viewed at 165,000× magnifications in an FEI Tecnai Arctica cryo-transmission EM operating at 200 kV and equipped with a Falcon 3EC direct electron detector.

### 4.5. Graphical Representation

Molecular representations were created using ChimeraX [68]. Other graphics were created using Adobe Illustrator (Adobe Inc., San Jose, CA, USA, 2019), BioRender (biorender.com, accessed 5 November 2022), and PowerPoint.

## Data Availability

Not applicable.
